# A Metabolic Smorgasbord Drives and Sustains CSC Heterogeneity

**DOI:** 10.3390/cancers15020343

**Published:** 2023-01-05

**Authors:** Michael Kahn

**Affiliations:** Department of Cancer Biology and Molecular Medicine, Beckman Research Institute of City of Hope, Duarte, CA 91010, USA; mkahn@coh.org

A deeper understanding of the biology of therapy resistance is important for the development of optimal strategies to attain complete cancer cures. Cancer stem cells (CSC) are the major cause of therapy resistance, associated with metastasis and the cause of disease relapse. However, normal somatic stem cells (SSC) and CSC share many common features, thereby complicating the safe elimination of CSC. Both SSC and CSC have the capacity to self-renew and differentiate, exist in different states (i.e., quiescent versus activated), and are plastic (i.e., can cycle between the different states).

A Smorgasbord is defined as a mixture of many different hot and cold dishes that are arranged so that you can serve yourself as much as you want in a buffet style. It is now clear that both normal stem cells (germ line and somatic) and CSC can sample many different “dishes” at the Smorgasbord table to suit their “behaviors” and “life-styles” by matching their metabolic needs. In their excellent review entitled “The Metabolic Heterogeneity and Flexibility of Cancer Stem Cells” Atsushi Tanabe and Hiroeki Sahara [1] discuss the metabolic preferences of cancer stem cells (CSC), analyze the flexible metabolic mechanisms of CSC and highlight therapeutics that may be able to target CSC metabolism. The authors also discuss the role of stromal cells in the tumor microenvironment including, endothelial cells, fibroblasts and adipocytes and how they provide various “dishes” including glucose, lactate, ketone bodies, fatty acids and glutamine to please the palates of different CSC via Smorgasbord dining. Interestingly, the authors also discuss the stage dependent roles of reactive oxygen species (ROS) in CSC and how quiescent CSC maintain low levels of ROS, which is generated via mitochondrial OXPHOS. ROS-induced damage to nuclear or mitochondrial DNA leads to genetic mutations in stem cell populations and therefore must be avoided in quiescent long-lived stem cell populations.

Tanabe and Sahara write that “CSCs have a peculiar metabolism that is distinct from non-CSCs to maintain their stemness properties” and furthermore that “CSCs can reprogram their metabolism to flexibly respond to environmental changes”. These statements are absolutely correct, however, even within the CSC population there is a significant degree of heterogeneity including quiescent and activated CSC, which affects their choice of favorite “dish” to dine on. There are a number of methods that have been used to define CSC including the expression of various markers (e.g., CD34, CD44, Lgr5, ALDH, CD133 etc.), functional properties (i.e., serial replating, engraftability into immunocompromised mice) and therapeutic resistance; however, they do not all define the same population of CSC. It has been demonstrated that multiple CSC populations with different growth and metastatic capacities exist simultaneously within a tumor [2,3]. However, there are conflicting hypotheses about whether CSC depend on glycolysis, mitochondrial metabolism, or other metabolic pathways for maintaining stem cell traits [4,5]. CSC-specific metabolism is also responsible for maintaining intracellular redox homeostasis by regulating metabolic balance and synthesizing antioxidants.

Mitochondrial fatty acid oxidation (FAO) has been found to be the major energetic contributor to the maintenance of the quiescent status of both SSC and CSC [6,7]. One interesting question regarding the use of FAO in quiescent stem cells (normal or cancerous) is how these cells avoid the generation of ROS when utilizing FAO, which would be deleterious to their genomic integrity and also cause their exit from quiescence. Very recently, this question was addressed in primordial oocytes, a long-lived, generally quiescent, stem cell population [8]. These cells need to maintain functionality and genomic integrity throughout and until the end of an organism’s reproductive life, i.e., in humans more than 40 years, to remain capable of producing healthy offspring. Primordial oocytes have relatively low mitochondrial activity with an absence of ROS generation. Primordial oocytes accomplish this via non-expression of mitochondrial complex I (MC1), which is responsible for the oxidation of NADH to NAD^+^ and the release of electrons, with inevitable leakage. The leaked electrons are received by oxygen to generate reactive ROS. How do primordial oocytes bypass their need for MC1? They do this by utilizing mitochondrial complex II, which catalyzes the oxidation of FADH2 to FAD2^+^ and also serves as an entry point for electrons. Although less efficient, this entry point can support ATP production, however without generating large amounts of ROS [9].

Interestingly, in our own recent study [10], we found utilizing sc-seq and sc-metabolic studies that the most deeply quiescent subset of an agnostically selected therapy resistant population of CD34^−^CD38^−^ chronic myeloid leukemia stem cells (LSC) utilize the same strategy as primordial oocytes to maintain their quiescent status. These most deeply quiescent LSC do not express MC1 complex genes, thereby allowing them to primarily utilize FAO while minimizing ROS damage. When these quiescent LSC are activated/or pharmacologically differentiated with a small molecule CBP/β-catenin antagonist, they start to re-express MC1 and change their “diet” and rely much more heavily on glycolysis. We believe that quiescent SSC, LSC and likely more generally CSC, rely on mitochondrial FAO, without complex I expression, thereby mitigating vulnerability to ROS. Quiescent CSC can thereby remain in G_0_ phase for many years making them highly resistant to cancer chemo- and immunotherapy [11,12] and a reservoir for disease recurrence and relapse. Limiting the ability of CSC to pick and choose their food from the “Smorgasbord table” may thereby prevent therapy resistance with the goal of safely providing complete cancer cures.

Importantly, we have previously demonstrated that specific small molecule CBP/β-catenin antagonists (i.e., ICG-001 or the clinical analog PRI-724) can safely eliminate CSC, even the most deeply quiescent subset, via forced symmetric differentiative divisions, without deleterious effect on long-lived normal quiescent SSC, which preferentially undergo asymmetric divisions [13,14]. This mechanism provides a means to safely differentiate away, both in vitro and in vivo, the most deeply quiescent subset of CML LSC [15]. Further investigations will be required to determine if this strategy can be extended more generally to quiescent therapy resistant CSC [14].
